# Integrative multi-omics analysis of dietary fibre-induced modulations in the composition and function of chicken caecal microbiota

**DOI:** 10.1038/s41522-026-00943-7

**Published:** 2026-02-25

**Authors:** Anum Ali Ahmad, Kellie Watson, Farina Khattak, Dominic Kurian, Rachel Kline, Sebastien Guizard, Laura Glendinning

**Affiliations:** 1 The Roslin Institute and Royal (Dick) School of Veterinary Studies, University of Edinburgh, Easter Bush Campus, Midlothian, UK; 2 Monogastric Science Research Centre, Scotland’s Rural College (SRUC), Edinburgh, UK

**Keywords:** Biotechnology, Microbiology

## Abstract

The sustainability of poultry farming faces significant challenges due to rising feed costs and competition with human food sources. Dietary fibre offers a promising, cost-effective alternative due to its beneficial impact on gut health. We utilised a multi-omics approach to understand the influence of soluble inulin and insoluble cellulose dietary fibres on the composition and function of caecal microbiota in broilers. High inulin supplementation (4%) significantly altered caecal microbial composition and promoted broader microbial metabolic adaptations, indicating a strong fermentative response to this soluble fibre source. In contrast, high cellulose (4%) had a minimal impact, reflecting its limited fermentability and structural complexity. These findings provide valuable insights into how different fibre types and quantities shape gut microbial communities and their functional potential. A deeper understanding of these interactions will aid in formulating targeted dietary strategies to optimise gut health, nutrient utilisation, and overall poultry performance.

## Introduction

Chickens (*Gallus gallus*) are the most consumed meat source worldwide, providing high-quality protein and micronutrients^1^. The poultry meat consumption is predicted to increase by 30% by 2033 due to a growing population, urbanisation, and rising incomes^2,3^ placing significant pressure on poultry production systems. The primary challenges for sustainable poultry production are feed cost and limited availability of conventional feed ingredients, such as maize, soy, and corn, which directly compete with the human food supply^4^. To address feed-food competition, alternative feedstuffs such as crops or agricultural by-products are being explored^5,6^. However, these feeds tend to be higher in dietary fibre, which presents a significant challenge for modern broiler chickens.

Recently, dietary fibre (NSP-non-starch polysaccharide) has become a major portion of chicken rations, fulfilling roughly 3-4% of the nutritional demands of the birds^7^. NSPs were initially thought to be anti-nutritive^8^, but recent studies have shown that soluble and insoluble components of NSP have distinct effects on poultry production performance^9^. Insoluble non-starch polysaccharides (NSP), including cellulose, are known to increase nutrient retention time and enhance feed efficiency, while soluble components (inulin, pectin, and glucan) are known to increase digesta viscosity and reduce nutrient absorption^7,10^. Chickens have a limited intrinsic ability to degrade fibre due to their short digestive tracts and lack of endogenous cellulolytic enzymes. Therefore, they are dependent on their gut microbiota to extract energy from NSPs present in fibrous feed ingredients.

Chicks raised in modern poultry settings lack maternal contact, which is crucial in shaping early gut microbiota^11^. As a result, the gut microbial community of commercially raised birds often lacks key microbial taxa involved in fibre fermentation^12^. This decreases the efficiency of fibre utilisation and limits growth performance. Targeted manipulation of the gut microbiota is an important strategy that can be performed to improve fibre fermentation in poultry.

Chicken caeca harbour the most complex microbial community in the chicken gut, making them the main site for fermenting NSP into volatile fatty acids (VFAs), which serve as an essential energy source for the host^13,14^. Studies have highlighted both the beneficial and adverse roles of dietary fibre in shaping gut microbiota and its potential impact on bird health through microbial manipulation^15–17^. To date, most poultry studies have utilised 16S rRNA sequencing approach to explore caecal microbial changes in response to NSP^17–19^. Studies employing shotgun metagenomics to gain deeper insights into microbial composition and functions are scarce and do not capture real-time gene expression or enzymatic activity^20^. As a result, it offers only a limited understanding of the metabolic processes underlying fibre fermentation. This study is the first to employ an integrative multi-omics approach, including metagenomics, metatranscriptomics, and metaproteomics, to explore the effects of inulin and cellulose dietary fibre on the caecal microbial composition and their functional profile in chickens. This study moves beyond descriptions of changes in the chicken gut microbiota upon fibre administration, allowing us to identify the key mechanistic drivers of chicken caecal fibre fermentation.

## Methods

### Experimental design and sampling

Animals were housed in premises licensed under a UK Home Office Establishment License within the terms of the UK Home Office Animals (Scientific Procedures) Act 1986 (Study number 71-2023: approved by the Roslin Institute Animal Welfare and Ethical Review Board Named Veterinary Surgeon and Named Animal Care and Welfare Officer). Housing and husbandry complied with the Code of Practice for Housing and Care of Animals Bred, Supplied or Used for Scientific Purposes.

### Experimental design

The study was conducted at the National Avian Research Facility, The Roslin Institute, University of Edinburgh, UK. Ninety chickens (1-day-old male Ross 308 s broilers) were sourced from a commercial hatchery (PD Hook LTD, Cote, Bampton). Birds were divided into five dietary groups in a completely randomised design. Birds were divided into five dietary groups, each consisting of six pens with three birds per pen in a completely randomised design. The five dietary groups were (1) control diet (CG): received a standard corn/soya-based diet with no added fibre; (2) grower A (IN1): received corn/soya-based diet supplemented with 1% inulin during both the starter and the grower phase; (3) grower B (IN4): received corn/soya-based diet supplemented with 1% inulin during starter phase and 4% inulin during the grower phase; (4) grower C (CE1): received corn/soya-based diet supplemented with 1% ARBOCEL during both the starter and grower phase; (5) grower D (CE4): received corn/soya-based diet supplemented with 1% ARBOCEL during starter phase and 4% ARBOCEL during grower phase.

All birds had free access to feed and water throughout the study period. The details of raw ingredients and nutritional composition of starter and grower diets across different dietary groups are presented in Supplementary Table 1. At day 35, one bird from each pen was randomly selected and euthanised via cervical dislocation for post-mortem caecal sampling for multi-omic analysis. The confirmation of death was performed through cessation of circulation.

The caecal contents (*n* = 30; 6 birds/group) from both caeca were removed and mixed. The homogenised content was then divided into 3 tubes: one tube containing 750 µl RNAlater for RNA analysis, and the other two separate tubes designated for DNA and protein analysis. Moreover, a section of mid-caecal epithelium was also excised and stored in a tube containing 750 µl RNAlater. The RNAlater-containing tubes were placed at 4 °C for 24 hours to allow RNAlater to permeate tissue and stabilise RNA by inhibiting RNase activity. The tubes were later stored at -80 °C until RNA isolation. The tubes for DNA and protein analysis were immediately snap-frozen on dry ice and stored at -80 °C until further analysis.

### Nucleic acid extraction and sequencing

Genomic DNA (*n* = 30) and total RNA (*n* = 30) were extracted from each homogenised caecal content using QIAamp PowerFecal Pro DNA Kit (QIAGEN®) and RNeasy PowerMicrobiome Kit (QIAGEN®) according to the manufacturer’s instructions, respectively. Extracted DNA samples were treated with RNase Cocktail™ Enzyme Mix (Thermo Fisher) at a 1:20 volume ratio and incubated at room temperature for 15-30 minutes to remove RNA contamination. Additionally, total RNA was isolated from caecal mid-epithelium using RNeasy® Mini kit (QIAGEN®) with a minor modification: a 5 mm stainless steel bead was added to each sample during pulverisation to enhance tissue lysis. DNA and RNA samples were purified using AMPure XP Beads (Beckman Coulter) at a modified 1:1 volume ratio, followed by the remaining steps as per the manufacturer’s instructions. For RNA samples, the beads were pre-treated with RNaseOUT™ Recombinant Ribonuclease Inhibitor (Thermo Fisher) according to the manufacturer’s instructions before the purification step. The quantity and quality of extracted nucleic acid were checked using Qubit® 4.0 (Invitrogen Life Technologies, Carlsbad, CA, USA) and agarose gel electrophoresis.

Shotgun metagenomics (DNA from caecal content), metatranscriptomics (total RNA from caecal content), and eukaryotic mRNA (total RNA from caecal mid-epithelium) sequencing of purified samples were performed by Novogene Corporation Inc using a NovaSeq Illumina platform, producing paired-end 150 bp reads. Metaproteomics analysis on caecal content was conducted by the Proteomics & Metabolomics Facility at The Roslin Institute, University of Edinburgh.

### Metagenomics data analysis

The shotgun metagenomics sequencing generated 12 Gb of raw reads per sample. The adapters and low-quality reads were removed using fastp (v0.23.4), and reads aligned to the chicken genome (release-111, bGalGal1.mat.broiler.GRCg7b, database downloaded 6^th^ June 2024) were removed using BWA MEM (v0.7.18)^21,22^. A de novo assembler, MEGAHIT (v.1.2.9), was used to generate single assemblies for each sample using the options --continue --kmin-1pass --k-list 27,37,47,57,67,77,87 --min-contig-len 1000 parameters^23^. The assembled contigs were indexed, and reads were mapped to assemblies using BWA MEM (v0.7.18). The resulting alignments were sorted and converted to BAM format using SAMtools-sort (v1.20), and summary statistics were generated using the command SAMtools-flagstat^24^. Coverage depth per contig was calculated using jgi_summarize_bam_contig_depths command from MetaBAT2 (v2.15)^25^. Metagenomic binning was subsequently executed using MetaBAT2, and the quality of the resulting Metagenome Assembled Genomes (MAGs) was assessed with CheckM2 (v1.0.1)^26^. The MAGs were dereplicated using dRep (v3.5.0) at 95% and 99% of Average Nucleotide Identity (ANI), and MAGs having completeness ≥80% and contamination ≤10% were filtered. However, MAGs dereplicated at 95% were used for downstream analysis to capture species-level diversity and reduce redundancy^27^. The taxonomic assignment of the MAGs was carried out with GTDB-tk using the classify_wf function with default parameters (v2.4.0, database downloaded 5^th^ August 2024), and abundance estimation was performed using CoverM with --min-read-percent-identity 95 and --min-read-aligned-percent 85 parameters (v0.7.0)^28,29^. The phylogenetic tree was constructed using phylophlan (v3.1.1) and rerooted using FigTree (v1.4.3) at the branch between the archaeal and bacterial MAGs. The rerooted tree was then visualized using iTOL v6 (https://itol.embl.de/).

### Metatranscriptomics data analysis

After assessing the quality of raw reads using fastQC (v0.11.7), FastP (v0.23.4) was used to remove poor-quality sequences and adaptors, while SortMeRNA (v4.3.6) was used to eliminate rRNA reads^30^. The clean reads were mapped to the chicken transcriptome (release-111, bGalGal1.mat.broiler.GRCg7b, database downloaded 31^st^ May 2024) from Ensembl (https://www.ensembl.org/index.html) using BWA MEM (v0.7.18) to remove host contamination, and SAMtools was used to obtain the unmapped reads^31^. Then, Megahit (v1.2.9) was utilised to generate single-sample assemblies. Unlike the metagenomic analyses, the minimum length was adjusted to 300 bp to capture smaller mRNAs. After indexing assemblies and mapping reads to assemblies using BWA MEM and generating summary statistics using SAMtools-flagstat, the feature counts within the coding DNA sequence region were quantified with HTSeq-count (v2.0.5)^32^.

MetaGeneMark (v.3.38) was used to identify coding regions from the assemblies, and Seqkit (v2.8.2) was used to produce statistics for those coding regions^33,34^. The DIAMOND (v2.1.8) database, built from UniRef90 FASTA (database download 8^th^ July 2024) and NCBI taxonomy files (download 9^th^ July 2024), was used to assign functional information to the coding regions^35^. The annotation results from DIAMOND and the feature count results from the HTSeq were used for downstream analysis. DIAMOND annotated genes were filtered based on bitscore >50, and were merged with HTSeq counts based on gene ID after removing any redundancy. Counts for genes encoding the same gene product were aggregated to reflect functional shifts in microbial communities, and the resulting dataset was used for expression analysis.

The phyloseq (v1.42.0) package in RStudio (v4.2.3) was used to calculate alpha (Shannon and ACE indices) and beta (Bray-Curtis distance) diversities based on TPM (Transcripts Per Million) values generated by CoverM^36^. The TPM values were transformed into raw count equivalents to make them suitable for ANCOM-BC2 (v2.0.3)^37^. Differential pairwise comparisons were then performed at the phylum, genus, and species levels using the Dunn test in ANCOM-BC2.

### Metatranscriptomics data analysis

The count data generated after processing the sequencing reads (Additional_file_1) were pre-filtered to retain rows with counts ≥ 10 in a minimum of 3 samples. The remaining counts were normalised using the variance stabilising transformation (VST) function implemented in DESeq2 (v1.38.3). The relative and differential expression analysis across different diet groups was conducted using DESeq2, and gene products with *p* ≤ 0.001 were included in the further analysis.

CAZyme annotation was performed by dbCAN3 (database built on 11^th^ July 2024) on coding sequences predicted by MetaGeneMark^38^. The dbCAN3 results included annotation from HMMER, dbCAN_sub, and DIAMOND databases. Enzymes annotated by at least two databases were included to explore the relative expression of CAZyme types and families. Moreover, GH and GT families were selected for differential expression analysis using DESeq2 (v1.38.3) due to their higher expression in this study and key role in carbohydrate metabolism.

### Linking MAGs to metatranscriptomics gene expression

The protein-coding regions within the MAGs were predicted using Prodigal (v2.6.3) and annotated using DIAMOND BLASTp^39^. The FASTA files of each MAG were indexed using BWA index, and quality-filtered metatranscriptomics reads from each sample were aligned to each MAG using BWA MEM. The mapped reads were used to generate BAM files using SAMtools-sort. Depth files were created to understand how many reads were mapped to each gene in MAGs using SAMtools-flagstat. Finally, HTSeq-count was used to generate gene counts by counting RNA reads aligned to annotated genes in MAGs using BAM files and the corresponding. gff annotation files generated by Prodigal. After merging gene counts with functional annotations from DIAMOND, read counts were normalised using estimateSizeFactors function of DESeq2. Genes and CAZymes that were significantly differentially expressed in our study were retained to compare their expression in IN4 and CE4 groups with the CG group in each MAG.

### Kraken2 analysis

A custom Kraken database was built (25^th^ June 2024) using microbial RefSeq genomes from NCBI (including bacteria, archaea, plasmids, viruses, fungi, plants, and protozoa), the chicken genome (bGalGal1.mat.broiler.GRCg7b), and GenBank assemblies from the NCBI BioProjects PRJNA715658, PRJEB64517, PRJEB33338, PRJNA543206, PRJNA377666, PRJNA668258, PRJEB57055 (data downloaded 16^th^ June 2024). Quality-controlled reads from metagenomic and metatranscriptomic sequencing were classified at the kingdom, phylum, and genus levels using Kraken2 (v2.1.3)^40,41^.

### Metaproteomics data analysis

Caecal contents were homogenised in an extraction buffer comprising 5% SDS, 6 M urea, and 50 mM triethylammonium bicarbonate (TEAB), pH 8.5, at a sample-to-buffer ratio of 1:10 (w/v). Homogenisation was performed using a Precellys homogeniser at 5000 × g for 20 seconds in a ceramic bead vial (Precellys Lysing Kit, Tissue Homogenising CK Mix). The homogenates were then centrifuged at 16,000 × g for 10 minutes, and the supernatant was transferred to low-binding protein vials. Samples were subsequently sonicated using a Bioruptor Pico Sonicator (Diagenode) for 10 cycles (30 seconds on / 30 seconds off per cycle). Following sonication, samples were centrifuged at 16,000 × g for 10 minutes. The resulting supernatant was collected, and protein concentration was determined using a BCA assay.

Tryptic digestion was carried out using S-Trap microcolumns (Protifi, USA), following the manufacturer’s protocol with minor modifications. In brief, 20 µg of protein in extraction buffer was reduced with 10 mM dithiothreitol at 37 °C for 1 hour and alkylated with 18.75 mM iodoacetamide at room temperature for 35 minutes in dark conditions. Alkylation was quenched by adding phosphoric acid to a final concentration of 1.25%, followed by the addition of six volumes of binding buffer (90% methanol in 100 mM TEAB). After gentle vortexing, the protein suspension was loaded onto an S-Trap microcolumn and centrifuged at 4000 × g for 1 minute. The column was then washed three times with 150 µL of binding buffer, each with a spin at 4000 x g for 1 minute. Proteolytic digestion was performed by adding 20 µL of digestion buffer (1 µg trypsin in 50 mM TEAB) and incubating the column at 47 °C for 2 hours. Peptides were sequentially eluted using 40 µL each of: (1) 50 mM TEAB, (2) 0.1% formic acid in water, and (3) 0.1% formic acid in 50% acetonitrile. Eluted peptides were pooled, cleaned using C18 stage tips, and dried under a vacuum desiccator.

Purified peptides were separated over a 70-minute gradient on an Aurora 25 cm column (IonOpticks, Australia) using an UltiMate RSLCnano LC system (Thermo Fisher Scientific) coupled to a timsTOF HT mass spectrometer via a CaptiveSpray ionisation source. The LC gradient was delivered at a flow rate of 200 nL/min, with a post-run washout step at 500 nL/min. Column temperature was maintained at 50 °C. Data-dependent acquisition (DDA-PASEF) was employed, with full MS scans acquired from 100 to 1700 m/z and ion mobility ranging from 1.45 to 0.65 Vs/cm² (1/K₀). Up to 10 PASEF MS/MS frames were acquired per cycle on ion-mobility-separated precursors, excluding singly charged ions, which were fully resolved in the mobility dimension. Intensity thresholds were set at 1750 counts (minimum) and 14,500 counts (target).

Raw mass spectral data were processed using MetaLab v1.1^42^, employing the embedded FragPipe (v23.0) algorithm^43^. Searches were conducted against the Chicken Gut MAG database (v1.0.1; 1322 species)^44^, supplemented with the UniProt chicken protein database (18,370 entries). MSFragger search parameters included a precursor and fragment mass tolerance of 20 ppm, trypsin enzyme specificity allowing up to two missed cleavages, and a minimum peptide length of seven amino acids. Carbamidomethylation of cysteine was specified as a fixed modification, and methionine oxidation was included as a variable modification. Differential expression analysis of the proteins was performed using DEP/limma, after filtering proteins with filter_missval() (threshold = 0) and identifying significant hits using an adjusted *P* < 0.05 and a log₂ fold-change of 1.5. A heatmap of proteins showing significant differences (*p* < 0.05) was plotted using the ggplot2 package.

### RNAseq analysis

The fastaq files of eukaryotic mRNA sequencing data were run through the nf-core/rnaseq (v3.14.0) pipeline using nextflow (v23.10.1)^45^. The reads were aligned against the chicken genome (bGalGal1.mat.broiler.GRCg7b) using STAR (v2.7.9a) and gene-level quantification was performed using Salmon (v1.10.1) (https://github.com/COMBINE-lab/salmon) to generate gene counts. Differential expression analysis was performed using DESeq2 (v1.38.3).

### Statistical analysis

Data normality was checked using the Shapiro-Wilk test. The significance of alpha diversity was analysed using the Kruskal-Wallis test, while the Dunn test was performed for multiple comparisons. Ordination analysis of Bray-Curtis distance was visualised using Principal Coordinate analysis. Permutational Multivariate Analysis of Variance (PERMANOVA) with 999 permutations was performed for microbial composition and function using the Adonis2 function from the vegan (v2.6.4) package. Graphs were visualised using ggplot2 (v3.5.0)^46^. The *P* values were adjusted for the false discovery rate using Benjamini-Hochberg method, and significance was defined as *p* ≤ 0.05.

## Results

### Metagenomic and metatranscriptomic data show differences in dominant microbial genera

**Fig. 1:**
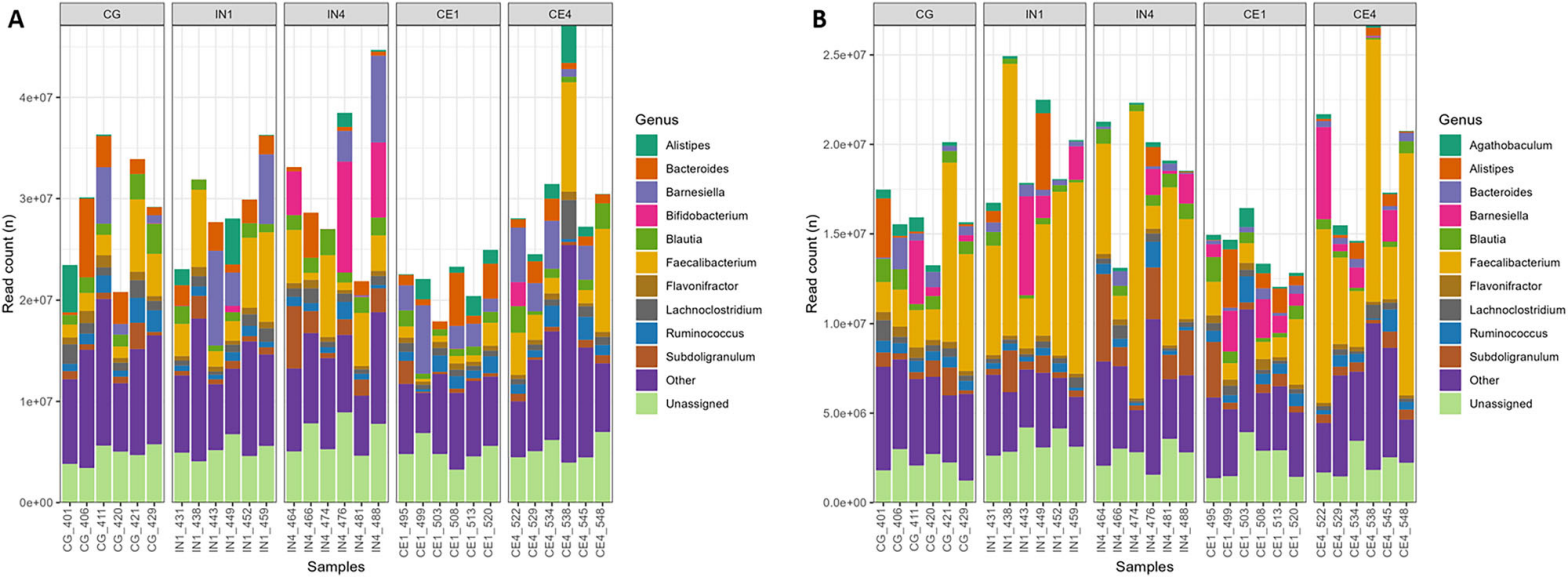
Kraken2 classification of metagenomic and metatranscriptomic reads. Metagenomics (**A**) and metatranscriptomics (**B**) reads classified at the genus level using Kraken2 across different dietary groups. Only the top 10 most abundant taxa are shown, while the remaining taxa are grouped as other. Reads that couldn’t be assigned any taxonomy are classified as unassigned. CG Control group, IN1 1% inulin, IN4 4% inulin, CE1 1% ARBOCEL, CE4 4% ARBOCEL.

Metagenomic (MG) and metatranscriptomic (MT) reads were classified at different taxonomic levels using Kraken2 to characterise the caecal microbial community (Fig. 1, Supplementary Fig. 1). This approach offers a comprehensive overview of microbial composition and their functional activity, enabling the identification of microbial taxa beyond bacteria and archaea, and providing insight into transcriptionally active members of the microbial community. Most reads were classified as bacteria (MG = 86.5% ± 0.67, MT = 89.3% ± 0.72), with some belonging to Archaea (MG = 0.21% ± 0.08, MT = 0.35% ± 0.14) and Eukaryota (MG = 0.42% ± 0.03, MT = 0.27% ± 0.03) (Supplementary Fig. 1, values represent mean%±SEM). Also, 13.4% ± 0.71 of the MG and 10.3% ± 0.70 of the MT reads were not assigned any taxonomy. Bacillota (MG = 65.4% ± 2.27, MT = 75.2% ± 1.83) and Bacteroidota (MG = 14.5% ± 1.84, MT = 10.2% ± 1.58) were the most classified phyla, while *Faecalibacterium* (MG = 11.7% ± 1.70, MT = 29.3% ± 3.65) and *Barnesiella* (MG = 8.05% ± 1.71, MT = 6.16% ± 1.49) were the most classified genera (Supplementary Fig. 1). We identified *Bifidobacterium* (2.33% ± 1.16) among the top 10 genera in the MG data, while it was replaced by *Agathobaculum* (1.85% ± 0.29) in the MT data (Fig. 1). This might be because *Bifidobacterium* was abundant but not very functionally active.

### 514 high-quality MAGs constructed from shotgun metagenome sequencing data

**Fig. 2:**
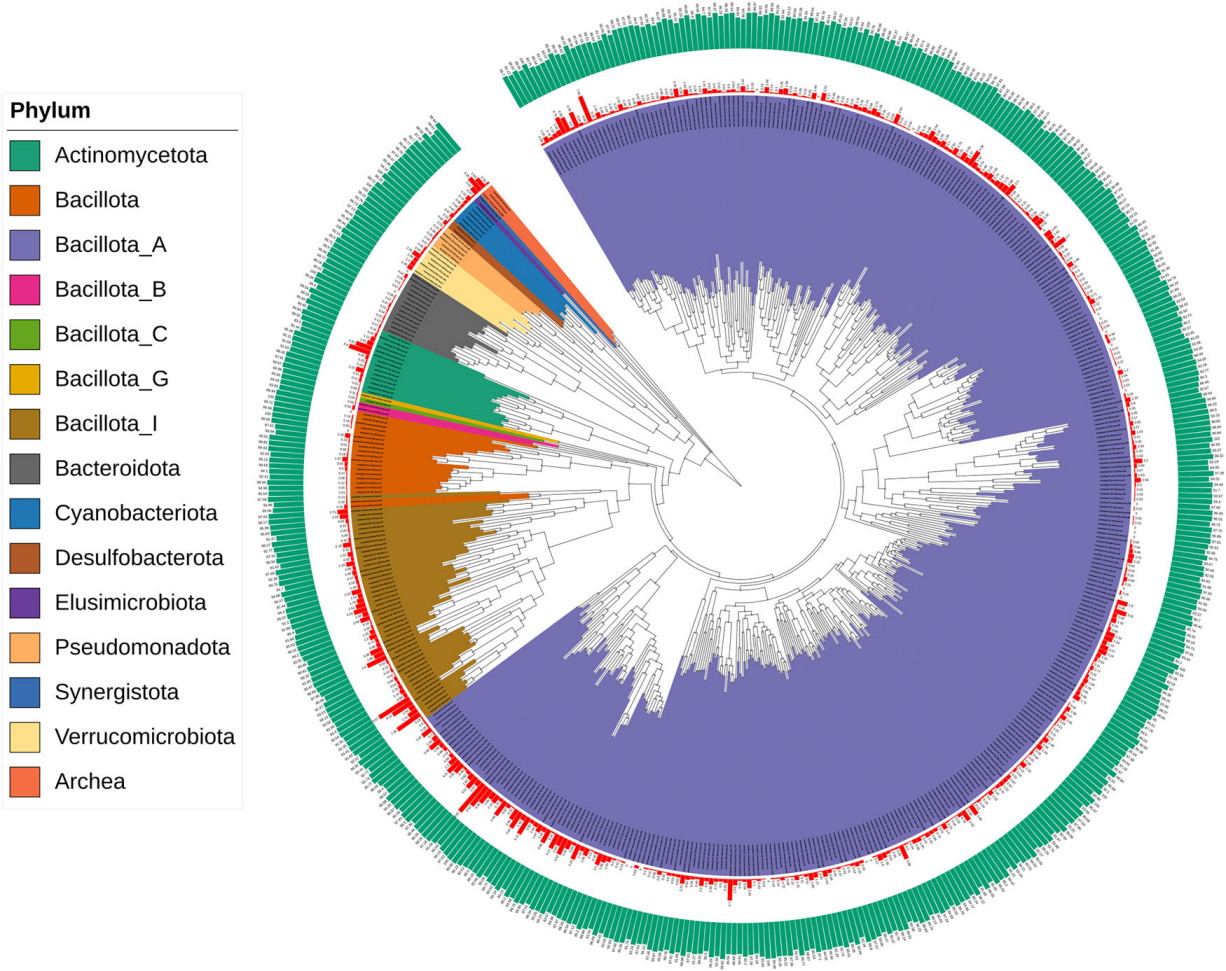
Microbial phylogenetic tree representing 514 metagenome-assembled genomes (MAGs) recovered from the caeca of chickens across all dietary groups. Background colours indicate the phylum to which MAGs belong. The red outer circle indicates contamination levels, while the green outermost circle represents completeness of MAGs.

We used metagenomic data to construct metagenome assembled genomes (MAGs), which allows the identification of unculturable, novel microbial species in the caeca. Shotgun metagenomic sequencing of caecal contents samples generated approximately 96.83 ± 3.03 million reads with an average length of 150 bp per sample. Clean reads (85.74 ± 3.34 million mean reads per sample) obtained after quality control and removal of host reads were assembled into 2,991,842 contigs with an average N50 value of 6699 bp. The contigs were binned into MAGs and dereplicated at 95% average nucleotide identity (ANI), a commonly used species-level threshold, to remove redundancy. The MAGs were then filtered to retain only those with contamination ≤5% and completeness ≥80%, resulting in the recovery of 514 high-quality MAGs. The details of each MAG are presented in Supplementary Table 3. Taxonomic annotation of MAGs revealed that 511 belonged to bacteria while 3 belonged to archaea (Fig. 2).

### High inulin quantity altered the caecal microbial diversity in chickens

**Fig. 3:**
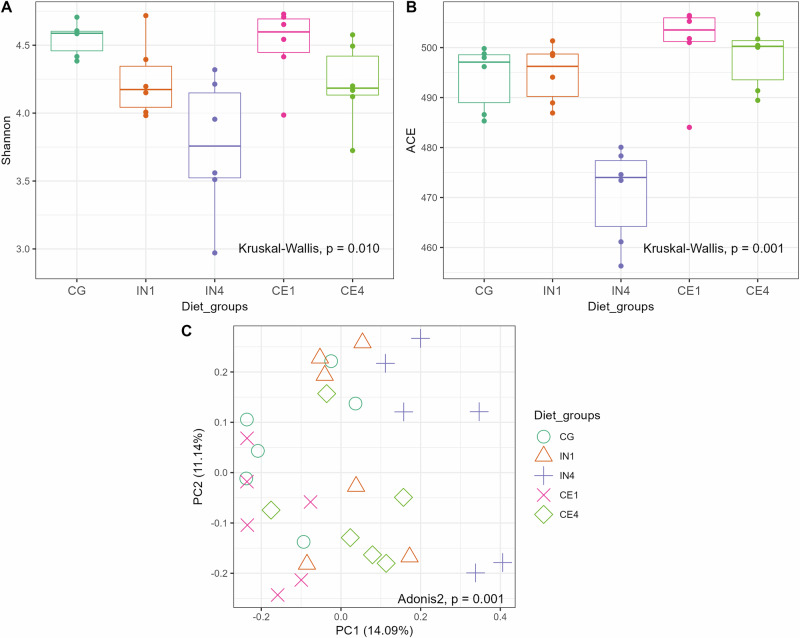
Influence of different quantities and types of dietary fibres on the diversity of caecal microbiota of chicken. Shannon (**A**) and ACE (**B**) indices showing evenness and richness of species across different dietary groups. The line inside each box represents the median value, the top and bottom lines indicate the 75th and 25th percentiles, respectively, while outliers, represented as dots, indicate values more than 1.5 times the interquartile range from the top or bottom of the box. Principal coordinate analysis (PCoA) of the caecal microbial community calculated by Bray-Curtis distance (**C**). Each symbol represents an individual sample, with the same symbols and colours indicating the same dietary group. Significance was defined as *p* ≤ 0.05. CG Control group, IN1 1% inulin, IN4 4% inulin, CE1 1% ARBOCEL, CE4 4% ARBOCEL.

We analysed caecal microbial diversity to assess the variety and abundance of microbial taxa in response to different dietary fibres. Alpha diversity analysis showed a significant effect of dietary fibre on the caecal microbial community of chickens. Microbial diversity measured by Shannon (Kruskal-Wallis, *p* = 0.01) and ACE indices (Kruskal-Wallis, *p* = 0.001) showed a decrease in the number of species and their relative abundance in the IN4 group compared to other dietary groups (Fig. 3A and B). Pairwise comparisons revealed significant differences in the IN4 group compared to the CG (*p* = 0.01) and CE1 (*p* = 0.01) groups using the Shannon index. Additionally, significant differences in the caecal microbial community of the IN4 group compared to the CE1 (*p* < 0.001) and CE4 (*p* < 0.01) groups were observed using the ACE index. Beta diversity analysis based on Bray-Curtis distance showed a significant difference among dietary groups (Adonis2, *p* < 0.001). Principal coordinates analysis (PCoA) revealed distinct clustering of samples from the IN4 group, indicating significant differences in their overall caecal microbial composition compared to other dietary groups (Fig. 3C). Pairwise PERMANOVA comparison showed that IN4 differed significantly from CG (R2 = 0.21, *p* = 0.02), CE1 (R2 = 0.24, *p* = 0.04), and CE4 (R2 = 0.15, *p* = 0.04) groups, while no significant differences were observed between other dietary groups.

### MAGs belonging to Bacillota_A dominated the chicken caecal microbial community

**Fig. 4:**
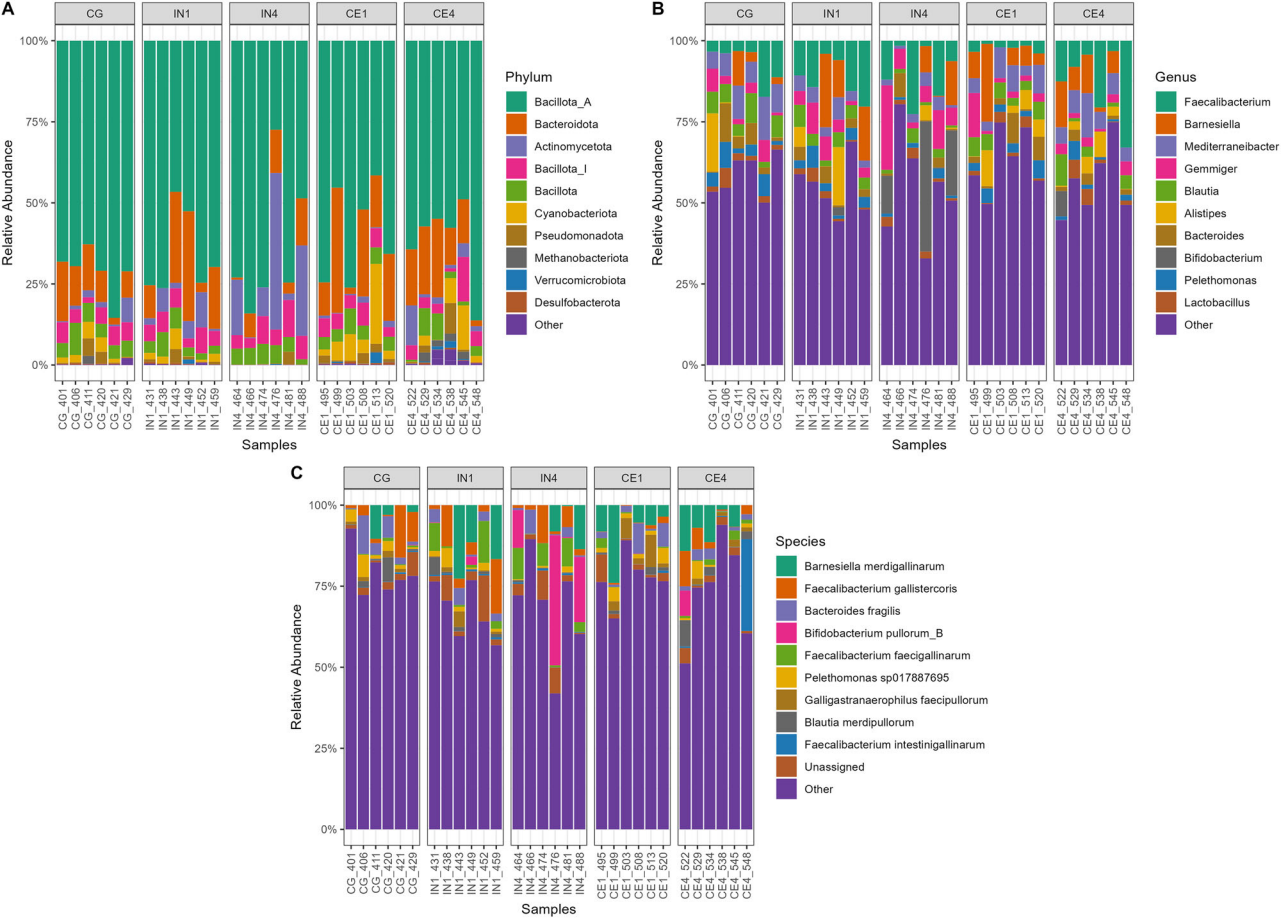
Influence of different quantities and types of dietary fibres on the abundance of caecal microbiota of chicken. The relative abundance of caecal microbiota of chickens at phylum (**A**), genus (**B**), and species (**C**) levels across different dietary groups. Each column denotes a sample. Only the top 10 most abundant taxa are shown, while the remaining taxa are grouped as other. The MAGs that couldn’t be assigned any taxonomy are grouped as unassigned. CG, Control group; IN1, 1% inulin; IN4, 4% inulin; CE1, 1% ARBOCEL; CE4, 4% ARBOCEL.

We identified highly abundant MAGs in our metagenomic data (Fig. 4). All the MAGs belonged to 15 phyla, out of which Bacillota_A (64.4% ± 2.61) and Bacteroidota (13.4% ± 1.85) showed the highest relative abundance across all dietary groups (Fig. 4A). Other major phyla included Actinomycetota (5.76% ± 1.84), Bacillota_I (5.37% ± 0.48), Bacillota (4.66% ± 0.41), and Cyanobacteriota (3.39% ± 0.93). Out of 259 genera, *Faecalibacterium* (8.96% ± 1.46), *Barnesiella* (5.88% ± 1.27), and *Mediterraneibacter* (5.56% ± 0.58) were highly abundant across all dietary groups (Fig. 4B). At the species level, MAGs belonging to *Barnesiella merdigallinarum* (5.88% ± 1.27), *Faecalibacterium gallistercoris* (4.02% ± 0.90), *Bacteroides fragilis* (2.86% ± 0.55), and *Bifidobacterium pullorum_B* (2.77% ± 1.51) were highly abundant across all dietary groups (Fig. 4C). MAGs (*n* = 26) that were not assigned any GTDB-TK taxonomy were grouped under Unassigned.

### High inulin quantity exhibited a strong modulating effect on the caecal microbiota

**Fig. 5:**
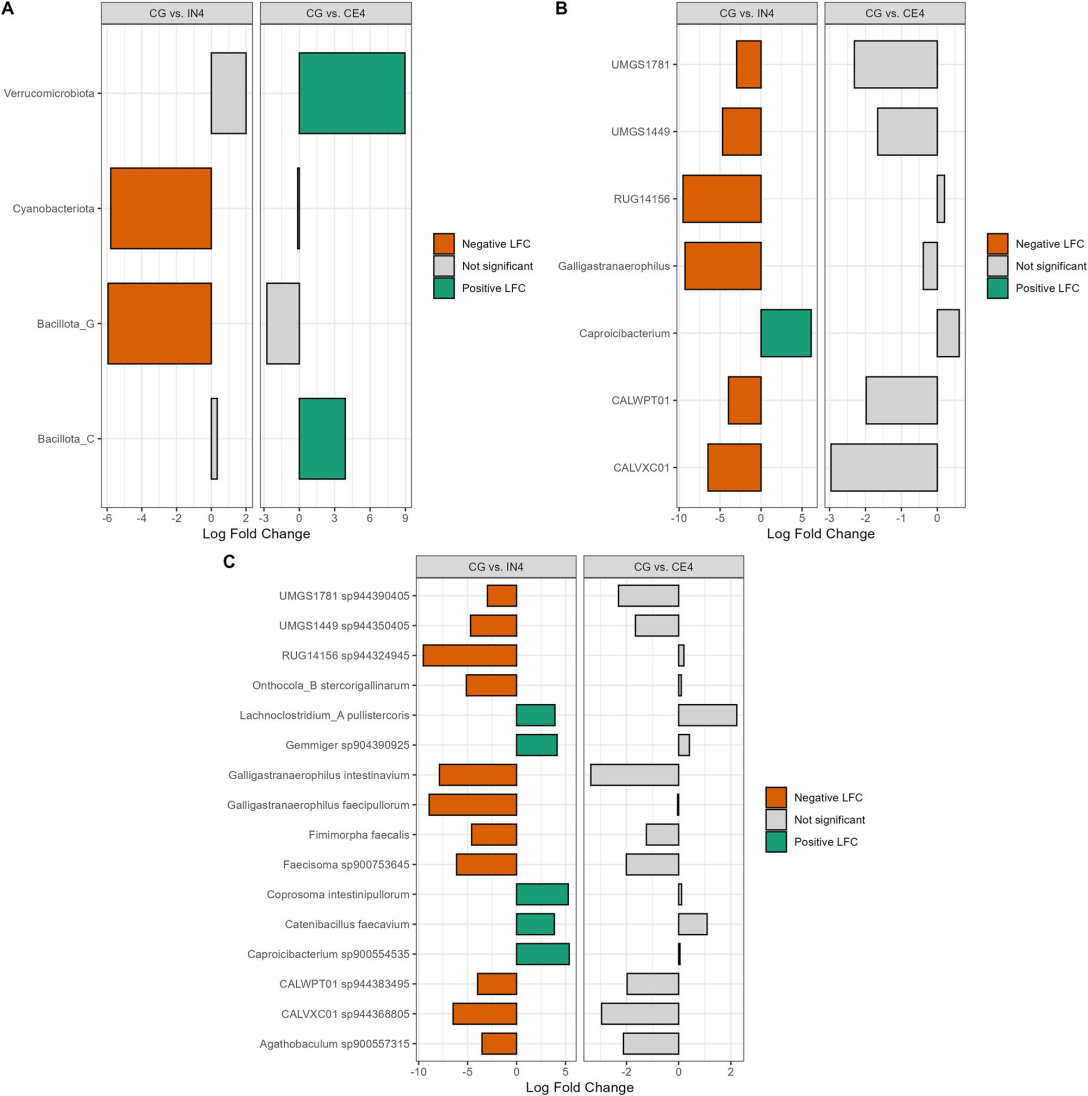
Influence of high dietary fibres on the caecal microbiota of chickens. The Bar graph shows the log fold change of significantly abundant caecal microbiota at the phylum (**A**), genus (**B**), and species (**C**) levels in pairwise comparisons: CG vs. IN4 and CG vs. CE4. A positive log fold change value (green) indicates a higher relative abundance in the latter group, while a lower relative abundance denotes the opposite (orange). Grey bars denote non-significant differences. CG Control group, IN1 1% inulin, IN4 4% inulin, CE1 1% ARBOCEL, CE4 4% ARBOCEL.

Differential abundance analysis was performed to explore the changes in the caecal microbial community of chickens. Since high inulin supplementation significantly affected the caecal microbial diversity in our data, we focused on high dietary fibre (4%) groups for further analysis. We performed pairwise comparisons of the CG group with IN4 and CE4 groups to explore the effect of high quantities of different dietary fibres on the caecal microbial community (Fig. 5). At the phylum level, Cyanobacteriota (*p* < 0.001) and Bacillota_G (*p* < 0.001) showed significantly lower relative abundance in the IN4 group compared to CG group. At the same time, Bacillota_C (*p* < 0.001) and Verrucomicrobiota (*p* < 0.01) were significantly more abundant in the CE4 group compared to the CG group (Fig. 5A). We observed changes in relative abundances of several genera and MAG in the IN4 group, showing a modulating effect of a high quantity of inulin on caecal microbial composition (Fig. 5B and C). At the genus level, *Caproicibacterium* (*p* < 0.001) showed significantly higher abundance in the IN4 group compared to the CG group (Fig. 5B). No significantly different genera were observed in CE4 compared to the CG group. For differential abundance analysis at the MAG level, we also included the MAGs that were not assigned any taxonomy and included only those present in more than 10% of our samples. MAGs *Lachnoclostridium_A pullistercoris* (*p* < 0.001), *Gemmiger sp904390925* (*p* < 0.001), *Coprosoma intestinipullorum* (*p* < 0.001), *Catenibacillus faecavium* (*p* < 0.001), and *Caproicibacterium sp900554535* (*p* < 0.001) were significantly more abundant in the IN4 group compared to the CG group (Fig. 5C). In contrast, we didn’t observe any significant differences between the CG and CE4 groups at the genus and MAG species levels. The pairwise comparisons between other dietary groups are presented in Supplementary Fig. 2.

### Lower expression of carbohydrate metabolism-associated genes in the high inulin group

**Fig. 6:**
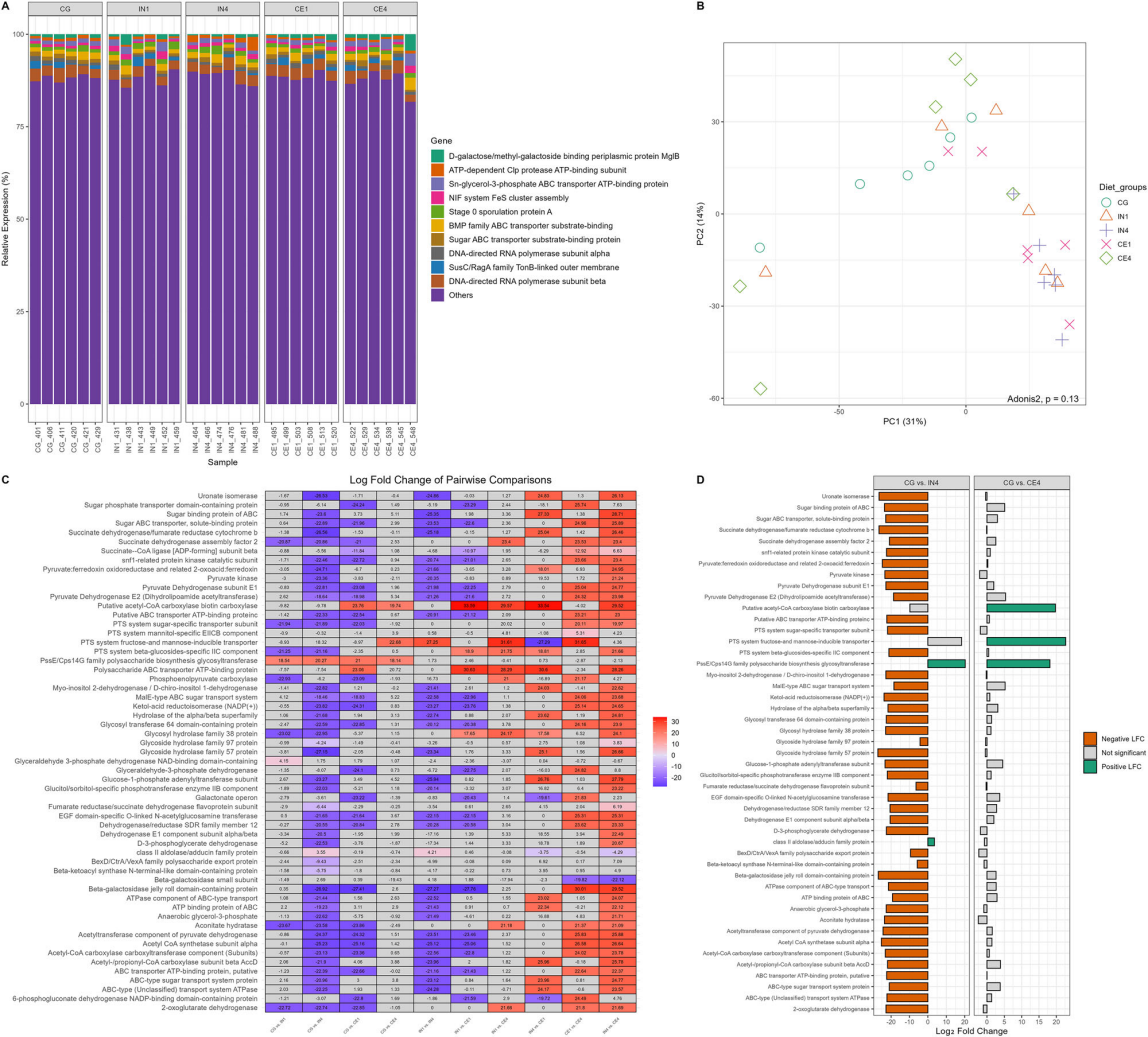
Influence of different dietary fibres on the functional profile of caecal microbiota of chickens. Principal coordinate analysis (PCoA) of the caecal microbial community calculated by Bray-Curtis distance (**A**). Each symbol represents an individual sample, with the same symbols and colours indicating the same dietary group. The relative gene expression of caecal microbiota of chickens across different dietary groups (**B**). Heatmap illustrates the log fold change of differentially expressed carbohydrate-associated genes in pairwise comparison of different dietary groups (**C**). The Bar graph presents a log fold change of significantly differentially expressed genes in pairwise comparisons, CG vs. IN4 and CG vs. CE4 (**D**). A positive log fold change value (green) indicates an upregulated expression in the latter group, while a downregulated expression denotes the opposite (orange). Significance was declared at *p* ≤ 0.05. Grey tiles/bars denote non-significant differences. CG Control group, IN1 1% inulin, IN4 4% inulin, CE1 1% ARBOCEL, CE4 4% ARBOCEL.

While metagenomics provides insights into microbial community composition and its potential functions, metatranscriptomics provides a snapshot of actively transcribed genes, offering insight into microbial metabolic activity. We used DIAMOND-annotated metatranscriptomics data to identify alterations in microbial gene expression in response to different dietary fibres. Relative expression analysis identified DNA-directed RNA polymerase subunit beta (2.83% ± 0.11) and BMP family ABC transporter substrate-binding (1.55% ± 0.11) to be highly expressed genes across all dietary groups (Fig. 6A, values represent mean%±SEM). Other relatively highly expressed genes included Sn-glycerol-3-phosphate ABC transporter ATP-binding protein (1.19% ± 0.13), Stage 0 sporulation protein A (1.15% ± 0.07, D-galactose/methyl-galactoside binding periplasmic protein MglB (0.92% ± 0.15), and ATP-dependent Clp protease ATP-binding subunit (0.91% ± 0.11).

Differentially expressed genes (DEGs) were identified to explore the effect of different types and quantities of dietary fibres on the functional profile of the chicken caecal microbiota. A total of 546 DEGs were identified across all dietary groups using DESeq2. A heatmap illustrating pairwise comparison of the top 30 DEGs across different groups is shown in Supplementary Fig. 3. Bray–Curtis dissimilarity analysis of normalised metatranscriptomics data using the variance stabilising transformation function revealed no significant differences in overall microbial gene expression profiles among dietary groups (Adonis2, *p* = 0.13). Ordination analysis further confirmed the absence of distinct clustering of samples by different dietary groups (Fig. 6B).

We specifically studied carbohydrate-associated DEGs to investigate the impact of different dietary fibres on the expression of these genes. A total of 57 carbohydrate-associated DEGs across all dietary groups were identified (Fig. 6C). We performed pairwise comparisons of DEGs from IN4 and CE4 groups with the CG group to understand the differences in microbial functional profiles in response to high quantities of different dietary fibres (Fig. 6D). Only two carbohydrate-associated DEGs were upregulated in the IN4 group. In the CE4 group, three DEGS, i.e., PssE/Cps14G family polysaccharide biosynthesis glycosyltransferase, putative acetyl-CoA carboxylase biotin carboxylase and PTS system fructose and mannose-inducible transporter subunit IIC were upregulated. We didn’t find any down-regulated DEGs in the CE4 group compared to the CG group. Overall, most of the DEGs involved in glycolysis, citric acid cycle, starch and glycogen biosynthesis, and glycoside hydrolase/glycosyltransferase activity showed lower expression in the IN4 group.

### High inulin quantity upregulated CAZyme families GH1 and GH32

**Fig. 7:**
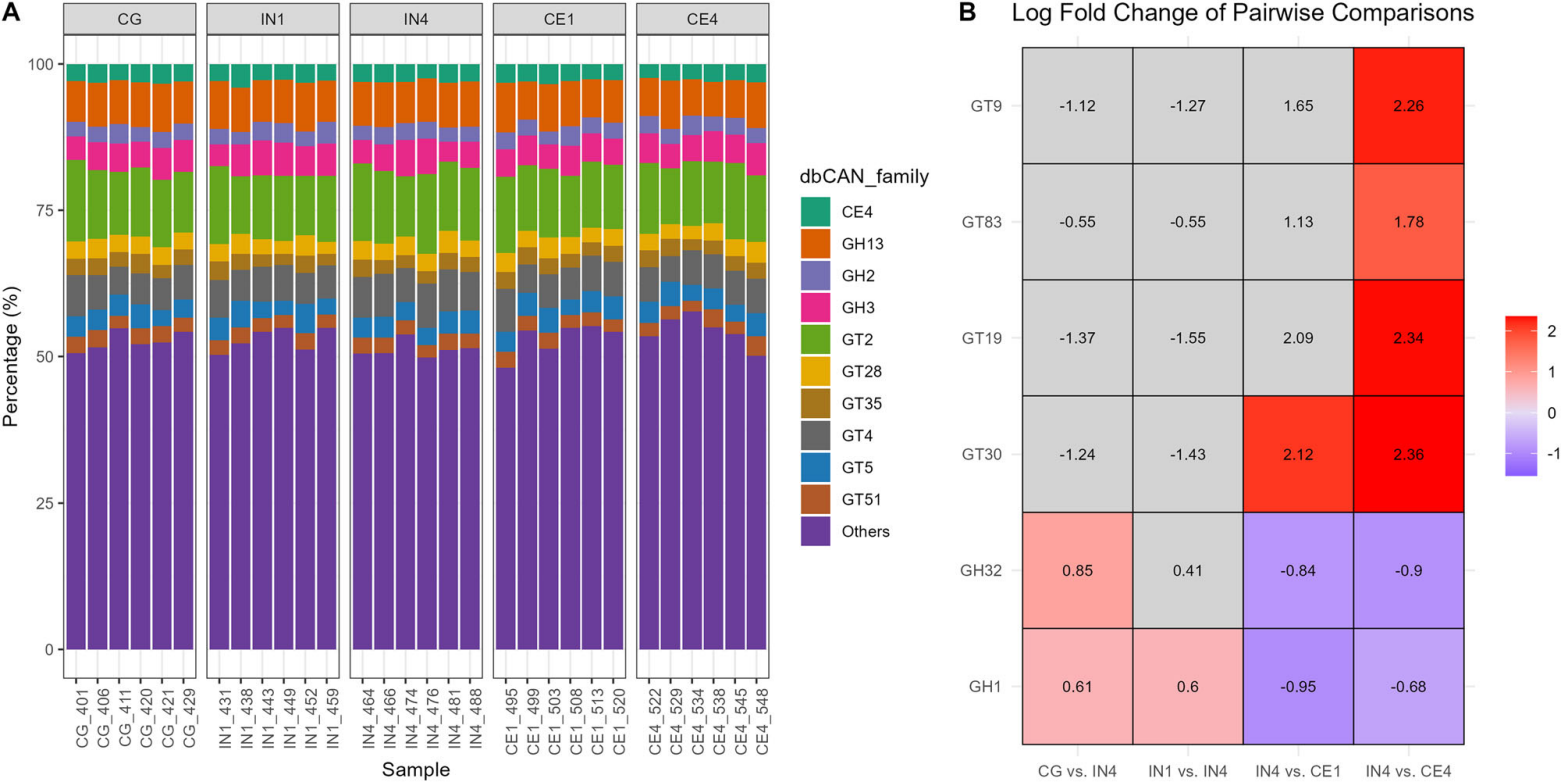
Influence of different dietary fibres on the CAZyme encoding genes across all the dietary groups. The bar graph shows highly expressed CAZyme families in the chicken caecal microbial community (**A**). Heatmap presents the log fold change of differentially expressed CAZyme families in pairwise comparison of different dietary groups, highlighting only significant results (**B**). Significance was declared at *p* ≤ 0.05. Grey tiles denote non-significant differences. CG Control group, IN1 1% inulin, IN4 4% inulin, CE1 1% ARBOCEL, CE4 4% ARBOCEL.

We next investigated carbohydrate-active enzymes (CAZYmes) genes, as they play a crucial role in enabling the gut microbiota to degrade and utilise dietary fibres. We identified a total of 119,269 CAZyme coding genes in our metatranscriptomics data using the dbCAN3 database. Glycoside hydrolases (GH) and glycosyltransferases (GT) were the most highly expressed CAZyme classes, accounting for 51.2% and 33.5%, respectively (Supplementary Table 2). GH is known to cleave glycosidic bonds to break down carbohydrates, while GT is involved in the formation of new glycosidic bonds to form or modify carbohydrate structures. Carbohydrate esterases (CE) class represented 7.7% and carbohydrate binding modules (CBM) represented 6.3% in our data. Some of the reads (0.17%) showed similarity to CAZymes, but could not be assigned to any known CAZyme class. This might be due to their low similarity to characterised CAZymes or limitations in the current CAZyme databases. Out of 552 CAZyme families identified in our data, GT2 (11.6% ± 0.21), GH13 (7.38% ± 0.12), GT4 (5.99% ± 0.14), and GH3 (4.89% ± 0.12) were highly expressed across all dietary groups (Fig. 7A, values represent mean%±SEM).

We then used the GH and GT classes to perform differential expression analysis due to their high relative abundance in our data and key roles in carbohydrate metabolism. Adonis2 analysis based on Bray-Curtis dissimilarity showed significant differences in GH (Adonis2, *p* = 0.001) and the GT (Adonis2, *p* = 0.01) classes. Ordination analysis showed separate clustering of CG and IN4 groups in GH class, while no clustering was observed in the case of GT class (Supplementary Fig. 4). Pairwise PERMANOVA comparison for the GH class showed significant differences in the IN4 group compared to CG (R2 = 0.21, *p* = 0.03) and CE1 (R2 = 0.24, *p* = 0.01) groups, while no significant differences were observed between other dietary groups. We didn’t find any pairwise significant difference between different dietary groups in the GT class.

We then used DESeq2 to perform pairwise comparisons between different dietary groups to identify significantly differentially expressed CAZyme families. A few of our pairwise dietary group comparisons showed significant differences. The upregulated expression of GH32 and GH1 families was found in the IN4 group compared to the CG group. The GH32 family enzymes, including inulinases and levanases, specifically break down inulin-type fructans into fermentable sugars. In contrast, the GH1 family includes β-glucosidases and β-galactosidases, which broadly hydrolyse β-linked oligosaccharides and glycosides in plant cell walls. The GT families, including GT9, GT83, GT19, and GT30, showed upregulation, and GH families, including GH32 and GH1, showed downregulation in the CE4 group compared to the IN4 group (Fig. 7B). No significant differences were observed between CE4 and CG groups for either family. These GT families are involved in the biosynthesis of various structural and functional glycoconjugates like lipopolysaccharides, exopolysaccharides, and glycoproteins. They transfer sugar moieties to various acceptors, supporting cell wall integrity, signalling, and interactions within microbial communities. Overall, the results suggest that the caecal microbiota in the CE4 group were more engaged in anabolic processes, whereas those in the IN4 group were more involved in sugar hydrolysis and degradation processes.

### MAGs showed a similar expression profile but different expression levels in different dietary groups

**Fig. 8:**
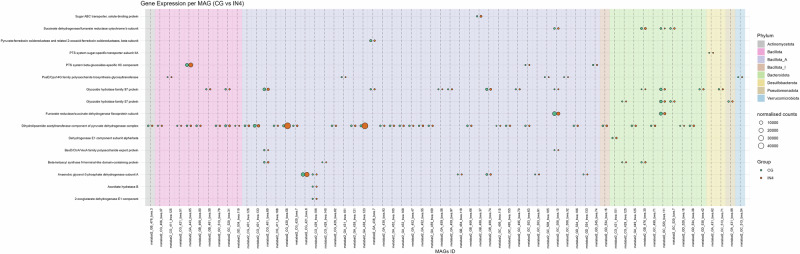
Bubble plots show the different expression levels of significantly differentially expressed carbohydrate-associated genes by different MAGs in IN4 compared to the CG group. Bubble size represents normalised counts per gene per MAG across different dietary groups. Background colour indicates the phylum to which each MAG belongs. CG Control group, IN4 4% inulin.

**Fig. 9:**
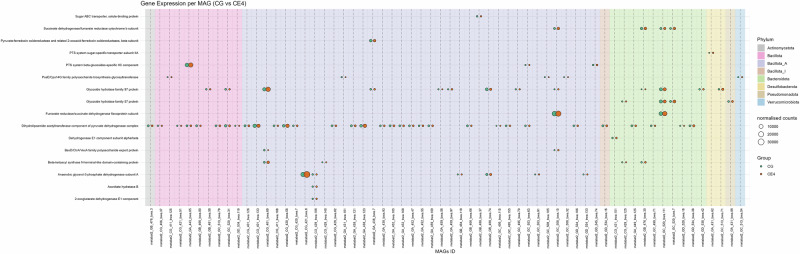
Bubble plots show the different expression levels of significantly differentially expressed carbohydrate-associated genes by different MAGs in CE4 compared to the CG group. Bubble size represents normalised counts per gene per MAG across different dietary groups. Background colour indicates the phylum to which each MAG belongs. CG Control group, CE4 4% ARBOCEL.

**Fig. 10:**
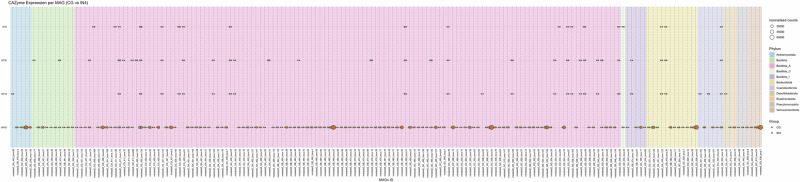
Bubble plots show the different expression levels of significantly expressed CAZymes by different MAGs in IN4 compared to the CG group. Bubble size represents normalised counts per gene per MAG across different dietary groups. Background colour indicates the phylum to which each MAG belongs. CG Control group, IN4 4% inulin.

**Fig. 11:**
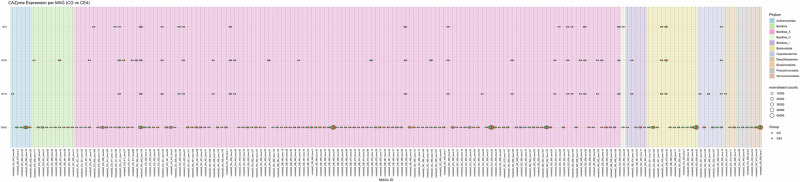
Bubble plots show the different expression levels of significantly expressed CAZymes by different MAGs in CE4 compared to the CG group. Bubble size represents normalised counts per gene per MAG across different dietary groups. Background colour indicates the phylum to which each MAG belongs. CG Control group, CE4 4% ARBOCEL.

We used MAGs and already identified significantly expressed CAZymes and carbohydrate-associated DEGs from our metatranscriptomics data to link them to their corresponding MAGs to find to what degree MAG genes are being expressed in different samples, thereby elucidating their roles in dietary fibre digestion. We compared the IN4 and CE4 groups with the CG group by using the significantly different DEGs and CAZymes data from our metatranscriptomics analysis (Figs. 8–11).

Out of 514 MAGs, 340 MAGs showed the expression of DEGs. We then filtered out genes which were expressed by 55% of the MAGs, including Glucose-1-phosphate adenylyltransferase subunit GlgD, Pyruvate kinase, and Uronate isomerase and ended up with 62 MAGs (Figs. 8 and 9). Most of the MAGs expressing DEGs belonged to Bacillota_A. Overall, the distribution of normalised gene counts across MAGs looked similar in CG vs. IN4 and CG vs. CE4 comparisons. However, group-specific differences in read counts were observed across dietary treatments for some MAGs. Gene counts for succinate dehydrogenase/fumarate reductase cytochrome b subunit by MAG metabat2_GC_520_bwa.111 (p_Bacteroidota;s_Alistipes megaguti) were lower in IN4 compared to the CG group, while no apparent difference was noted between the CE4 and CG groups. Higher gene counts for PssE/Cps14G family polysaccharide biosynthesis glycosyltransferase were observed in the CG group compared to IN4 and CE4 groups by different MAGs, including metabat2_GA_431_bwa.101 (p_Bacillota_A;s_Flemingibacterium merdigallinarum), metabat2_GA_452_bwa.105 (p_Bacillota;s_Scatomorpha sp900759385), and metabat2_GC_508_bwa.32 (p_Bacillota_A;s_Scatomorpha sp900545405), while an opposite trend was observed by MAG metabat2_CG_411_bwa.125 (p_Bacillota;s_Limosilactobacillus oris). These results indicate specific functions performed by different MAGs in the digestion of dietary fibre in the caeca of chicken. The details of other DEGs showing higher gene counts in IN4 and CE4 groups compared to the CG group are presented in Supplementary Table 4.

We then analysed the gene counts for CAZymes by the MAGs (Figs. 10 and 11). For this, we used CAZymes identified as significantly differentially expressed in our metatranscriptomics analysis to understand the contribution of individual MAGs in their expression. Overall, a total of 349 MAGs were involved in the expression of significantly differentially expressed CAZymes across dietary groups. GH1 and GT83 were expressed by ≥80% of all the MAGs, which were removed for downstream analysis, leaving 176 MAGs for further study. The distribution of gene counts for CAZymes, including GH32, GT19, GT30, and GT9, was visualised in CG vs. IN4 and CG vs. CE4 comparisons. Just like the DEGs, the overall distribution of normalised CAZyme gene counts was similar across all dietary groups, but group-specific differences were observed for some MAGs. Higher gene counts of the GH32 family were observed in IN4 and CE4 groups compared to the CG group by different MAGs. The details and taxonomies of MAGs are presented in Supplementary Table 3. The gene counts for GH32 were lower in IN4 and higher in CE4 group compared to CG group by metabat2_CG_420_bwa.139 (p_Bacillota_A;s_Scatosoma pullicola). We observed higher gene counts for GT30 in the CE4 group compared to the CG group by MAGs (Supplementary Table 4); however, no differences were observed between the IN4 and CG groups.

### Dietary fibres showed minimal influence on the carbohydrate-associated protein expression of caecal microbiota

We performed metaproteomics analysis to understand which proteins were produced by caecal microbiota in response to different dietary fibres. A total of 48554 unique proteins were identified, reduced to 6996 after filtering for proteins quantified in all replicates of at least one group. We identified 63 differentially expressed proteins (DEPs) through pairwise comparison between different dietary groups, out of which only five DEPs were carbohydrate-associated (Supplementary Fig. 5). Pairwise comparison of IN4 and CE4 with the CG group showed higher relative expression of glyceraldehyde-3-phosphate dehydrogenase A and lower relative expression of alpha-galactosidase in the CE4 group, while higher relative expression of pyruvate, phosphate dikinase, and lower relative expression of pyruvate oxidase were observed in the IN4 group (Supplementary Fig. 6).

### Dietary fibres showed minimal effect on the expression of immune-associated genes in the host caecal epithelium

Dietary fibre is known to influence the immune system of the host through gut microbiota. Therefore, we explored immune-associated DEGs across all dietary groups (Supplementary Table 5). We did not identify any DEGs related to the immune system in the IN1 and CE1 groups compared to the CG group. However, we identified downregulations of two immune-associated DEGs i.e., *CCR8L* and *S100A10*, in the IN4 groups, while three immune-associated DEGs, i.e., *GPC3*, *FOXN1*, and *EFNA2*, showed upregulations in the CE4 group compared to the CG group. The majority of DEGs lacked functional annotation or were assigned to hypothetical proteins, making it challenging to interpret the results.

## Discussion

Dietary fibre is incorporated into poultry rations as an alternative feed resource to improve gut health, reduce feed-food competition, and promote sustainable production. Dietary fibre, once thought to be anti-nutritional^8^, is now recognised for supporting immunity, modulating gut microbiota, and improving digestive efficiency^47,48^. The chicken caecum is the main site of microbial fermentation, harbouring a complex microbial community involved in fermenting dietary fibre to extract energy from otherwise indigestible components. Inulin and cellulose are two contrasting fibre sources with distinct physicochemical and fermentative characteristics. Inulin is a type of fructan prebiotic that promotes the growth of beneficial gut microbiota^49^. While cellulose is an insoluble polysaccharide that enhances gut health and improves nutrient utilisation by supporting microbial fermentation^50^. Insights into how these dietary fibres shape gut microbiota are key to improving poultry production through targeted microbiota manipulation. In this study, we examined the effects of soluble inulin and insoluble cellulose dietary fibres on the composition and functional potential of caecal microbiota in broiler chickens using a multi-omics approach.

We compared the taxonomic assignments of metagenomic and metatranscriptomic reads in response to different dietary fibres to identify which taxa are abundant and which are functionally active. Among the top ten genera identified, we observed higher read counts of *Bifidobacterium* in metagenomic data, which *Agathobaculum* replaced in metatranscriptomics data. This difference highlights that high microbial abundance does not necessarily reflect metabolic activity and functionally active taxa might be underrepresented in DNA-based profiles. Another study reported similar findings, where several low-abundance genera showed metabolic activity in response to dietary fibre supplementation^15^. These findings underscore the value of integrating metatranscriptomics to complement taxonomic profiling and better capture gut microbial functions.

In this study, high inulin quantity significantly reduced the microbial diversity, which is inconsistent with previous reports showing that inulin increased microbial diversity^51^. This discrepancy might be due to the high inulin concentration (4%) in our study compared to 1.2% previously used. Based on these distinct changes, we focused on the high fibre groups to explore how increased dietary fibre shaped the caecal microbial community in chickens. We found that high inulin had a stronger influence on caecal microbial community than high cellulose, with significant variations observed at phylum, genus, and MAG levels. Previous studies reported an increase in the genus *Bifidobacterium* and *Lactobacillus* in response to inulin^52–54^. However, in our study, *Caproicibacterium* showed significantly higher abundance in the IN4 group. Although *Bifidobacterium* and *Lactobacillus* were highly abundant in the inulin groups, they didn’t show significant differences compared to the control group, consistent with previous findings^15^. This suggests that high inulin favoured the growth of alternative fibre-degrading or fatty acid-producing taxa such as *Caproicibacterium*^55^, which might be able to utilise inulin-derived substrates under high fibre conditions. On the other hand, the influence of high cellulose quantity was minimal on microbial composition, with significant variations primarily limited to the phylum level only. This aligns with previous findings that lignocellulose supplementation didn’t modify the ileal microbial composition in young broilers^56^. These differences likely stem from the recalcitrant nature of cellulose^57^, which is harder for microbes to degrade compared to the more fermentable inulin. This highlights the significance of fibre type, suggesting that soluble fibre might be more effective in modulating the caecal microbiota of broilers.

Analysis of the microbial functional profile revealed a decreased expression of genes associated with core metabolic processes, such as glycolysis, citric acid cycle, starch, and glycogen biosynthesis in the IN4 group. Similar findings were reported in the caecal microbiota of Salmonella-infected chickens supplemented with inulin^52^. Moreover, lower expression of pyruvate oxidase and higher expression of pyruvate, phosphate dikinase (PPDK) proteins were also noted. These enzymes are involved in pyruvate metabolism and work through distinct metabolic pathways. Pyruvate oxidase facilitates acetate production via pyruvate decarboxylation^58^, while PPDK supports gluconeogenesis and energy conservation by reversing glycolysis under anaerobic conditions^59^. These shifts suggest a broader metabolic adaptation by the microbial community, shifting from central energy metabolism to more specialised fermentative processes tailored to the inulin-rich environment. These functional changes were further supported by significantly higher expression of GH32 and GH1 families in the IN4 group. GH32 encodes inulinase, an enzyme that hydrolyses inulin-type fructans into fermentable sugars^60^, while GH1 encodes β-glucosidases, which cleave glycosidic bonds in oligosaccharides and contribute to the final steps of carbohydrate degradation^61^. Moreover, most of our MAGs showed expression of GH1 gene, indicating its functional importance within the caecal microbial community. The increased expression of these CAZyme genes suggests that microbes not only utilise readily available inulin through GH32 activity but also show broader adaptation to secondary β-glucosidic substrates released during microbial fermentation. In contrast, the functional changes in caecal microbiota in response to high cellulose were more subtle. We observed higher expression of three carbohydrate-associated genes linked to sugar transport, fatty acid synthesis, and polysaccharide biosynthesis, along with elevated enzyme levels of glyceraldehyde-3-phosphate dehydrogenase A. It is a key glycolytic enzyme^62^, indicating increased glycolytic activity to support basal energy demand with limited fermentable substrates. These findings suggest that microbes in the CE4 group preferred corn/soy components, as cellulose is challenging to break down and extract energy through glycolysis and the TCA cycle for fatty acid production, and perform biosynthetic activity to maintain cellular structure. Caecal microbes exhibit distinct functional adaptations to different dietary fibres. While inulin promotes sugar degradation and enzyme expression, cellulose tends to elicit more structural or regulatory responses, likely due to its recalcitrance and lower fermentability. These underscore the importance of fibre type and quantity in shaping not only microbial composition but also metabolic function within the gut ecosystem.

This study is the first to use multi-omics approaches to simultaneously characterise microbial composition and their functions at the transcript and protein levels in relation to fibre fermentation in chickens. The results reveal how different fibre types influence not only microbial composition but also their active metabolic roles. Ultimately, this research lays the groundwork for developing targeted, fibre-based dietary strategies to enhance gut health, nutrient utilisation, and productivity in poultry. Despite these insights, several limitations should be noted. First, sampling was performed at a single time point, which may not capture temporal shifts in microbial dynamics. Second, both shotgun metagenomic and metatranscriptomic analyses rely on existing reference databases, which may underrepresent certain microbial taxa or genes, limiting resolution. Third, although baseline oil levels were used in this study, fibre-fat interactions from fat supplementation to balance energy in fibre-rich diets should be noted due to their impact on caecal microbiota^63,64^. Finally, microbial functions were characterised at the transcript and protein levels; however, downstream outcomes, such as short-chain fatty acid production and host responses, were not assessed. These aspects warrant further investigation to elucidate the implications of dietary fibre interventions in poultry.

In conclusion, this study provides insights into how soluble (inulin) and insoluble (cellulose) dietary fibres modulate the composition and functions of the caecal microbiota in broiler chickens. Using a multi-omics approach, we found that high inulin quantity significantly altered microbial diversity, enhanced GH32 gene expression, and shifted metabolism away from central energy pathways, indicating microbial adaptation to fermentable fibre. In contrast, high cellulose had minimal influence on microbial composition but enhanced glycolytic activity to meet basic energy needs of microbes. These findings highlight the importance of fibre type and quantity in shaping gut microbiota and their functions, with potential implications for improving feed efficiency, nutrient utilisation, and poultry health.

## Supplementary information

Additional_file

## Data Availability

The datasets generated during and/or analysed during the current study (fastq files, assemblies, genome bins, and metagenome assembled genomes) are available in the European Nucleotide Archive under the project number PRJEB77488 (https://www.ebi.ac.uk/ena/browser/view/PRJEB77488). Metaproteomics data are available via ProteomeXchange with identifier PXD068444 (https://www.ebi.ac.uk/pride/archive/projects/PXD068444).
